# Temperature-dependent electronic structure of bixbyite α-Mn_2_O_3_ and the importance of a subtle structural change on oxygen electrocatalysis

**DOI:** 10.1080/14686996.2020.1868949

**Published:** 2021-04-09

**Authors:** Junais Habeeb Mokkath, Maryam Jahan, Masahiko Tanaka, Satoshi Tominaka, Joel Henzie

**Affiliations:** a International Center for Materials Nanoarchitectonics (WPI-MANA), National Institute for Materials Science (NIMS), Tsukuba, Japan; b Department of Physics, Kuwait College of Science and Technology, Kuwait; c Synchrotron X-ray Station at SPring-8, National Institute for Materials Science, Sayo, Japan

**Keywords:** Manganese oxide, Jahn–Teller distortions, phase change, OER, ORR, 401 1st principles methods, 504 X-ray / Neutron diffraction and scattering, 207 Fuel cells / Batteries / Super capacitors, 302 Crystallization / Heat treatment / Crystal growth

## Abstract

Bixbyite α-Mn_2_O_3_ is an inexpensive Earth-abundant mineral that can be used to drive both oxygen evolution (OER) and oxygen reduction reactions (ORR) in alkaline conditions. It possesses a subtle orthorhombic → cubic phase change near room temperature that suppresses Jahn–Teller distortions and presents a unique opportunity to study how atomic structure affects the electronic structure and catalytic activity at a temperature range that is easily accessible in OER/ORR experiments. Previously, we observed that heat-treated α-Mn_2_O_3_ had a better performance as a bifunctional catalyst in the oxygen evolution (OER) and oxygen reduction reactions (ORR) (*Dalton Trans*. 2016, 45, 18,494–18,501). We hypothesized that heat-treatment pinned the material into a more electrochemically active cubic phase. In this manuscript, we use high-resolution X-ray diffraction to collect the temperature-dependent structures of α-Mn_2_O_3_, and then input them into ab initio calculations. The electronic structure calculations indicate that the orthorhombic → cubic phase transition causes the Mn 3*d* and O 2*p* bands to overlap and mix covalently, transforming α-Mn_2_O_3_ from a semiconductor to a semimetal. This subtle change in structure also modifies Mn-O-Mn bond distances, which may improve the activity of the material in oxygen electrochemistry. OER and ORR experiments were performed using the same electrode at various temperatures. They show a jump in the exchange current density near the phase change temperature, demonstrating the higher activity of the cubic phase.

## GRAPHICAL ABSTRACT

**Figure:**
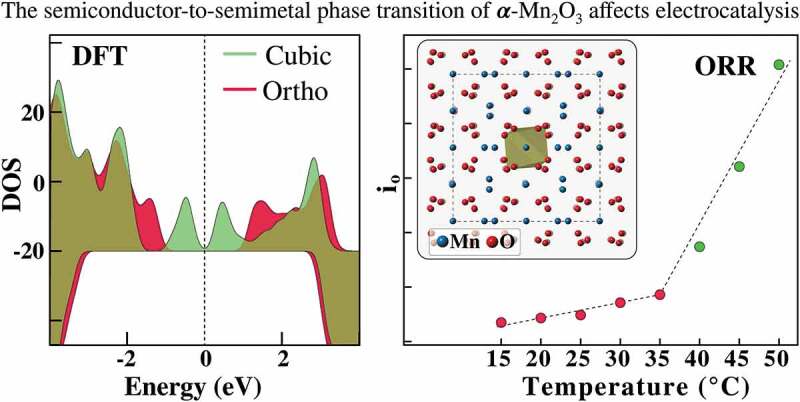

## 1. Introduction

Electrochemical energy generation and storage devices, including fuel cells [1], water-splitting systems [2,3], electrochemical capacitors [4], and rechargeable batteries [5,6] require inexpensive electrodes to efficiently drive electrochemical reactions. Precious metal-based electrocatalysts are currently favored in these technologies because they have superior performance and stability, but their high cost and scarcity limits feasibility for large-scale adoption. Researchers have been searching for more Earth-abundant transition metal catalysts that can deliver acceptable performance at extremely low economic cost. Transition metal oxide-based minerals have emerged as an inexpensive alternative due to their relatively high abundance in the Earth’s crust [7,8]. Manganese(III) oxides such as α-Mn_2_O_3_ contain Mn^3+^ metal cations with surface Mn^3+^O_6_ octahedral sites with one electron in the e_g_ orbital and moderately conductive metal-oxygen bonds. It has previously been reported that trivalent manganese oxides with Jahn–Teller distorted octahedra and an anti-bonding e_g_^1^ electron have high activity for the oxygen evolution reaction (OER) [9], enabling reversible interactions with O_2_ molecules that contribute to structural flexibility during water oxidation [10,11]. Interestingly, α-Mn_2_O_3_ electrodes can also drive the oxygen reduction reaction (ORR) in the same medium in a bifunctional electrocatalytic setup [12,13]. In situ X-ray results indicate that the Mn(III) active sites of the manganese oxide form a mixed Mn^II^/Mn^III^ state under OER conditions and a mixed Mn^III^/Mn^IV^ state under ORR conditions [14].

There are numerous transition metal oxide catalysts with higher OER and ORR performance compared to α-Mn_2_O_3_ [15–17]. But α-Mn_2_O_3_ provides an interesting case study to examine how subtle changes in atomic structure affect the Mn^III^ active site because it has an orthorhombic → cubic phase change at 300 K(26.85°C) [18]. This phase change is accompanied by a suppression in Jahn–Teller distortions in the cubic phase. And it has been shown that suppression of Jahn–Teller distortions in some manganese oxide systems increases OER catalytic activity [19]. Thus α-Mn_2_O_3_ presents a unique opportunity to study how suppression of Jahn–Teller distortions affects electronic structure and catalytic activity within a temperature range that is easily accessible in OER/ORR experiments. Previously, we reported a colloidal method to generate phase-pure α-Mn_2_O_3_ prisms bound with {100} facets by exploiting the cation bridging effect between Mn ions and sodium docusate [20]. Pair distribution function (PDF) X-ray analysis showed the as-prepared material was orthorhombic phase but it could be pinned in the cubic phase by thermally-treating α-Mn_2_O_3_ powder on a glassy carbon electrode (GCE) in air at 480°C. The heat-treated α-Mn_2_O_3_ prisms possessed far better bifunctional OER/ORR performance compared to the as-prepared prisms. We hypothesized that the improvement in performance was at least in part due to the phase transition, but other factors are always at play in catalyst experiments due to different electrode preparation methods, which may increase the surface area of the catalyst or improve contact between the α-Mn_2_O_3_ and the GCE support.

In this manuscript, we describe our efforts to study how electronic structure is affected by the orthorhombic → cubic phase change, and ultimately how this subtle structural change affects oxygen electrocatalysis. Temperature-dependent high-resolution X-ray diffraction (XRD) synchrotron measurements were used to obtain the precise orthorhombic and cubic crystal structures at various temperatures below and above the phase transition temperature. These structures were analyzed with the Rietveld refinement method to identify the different crystal systems with exceptional goodness of fits (GOF < 1.82) for a powder system. The resulting orthorhombic and cubic crystal structures were then used as models in density functional theory (DFT) simulations to examine how electronic band structure changes around the phase transition temperature. The DFT results indicate that the phase change causes α-Mn_2_O_3_ to transform from semiconductor → semimetal due to the suppression of some Jahn–Teller distortions. Then, OER/ORR experiments in a temperature-controlled electrochemical setup were used to examine how the α-Mn_2_O_3_ orthorhombic → cubic phase transition affected the exchange current density using the same electrode. Temperature-dependent conductivity measurements on the powder show that the electrochemically active surface area (ESCA) of the material does not change over the temperature range of the experiments, so we concluded that the marked increase in exchange current density in OER/ORR is due to the orthorhombic (semiconductor) → cubic (semimetal) phase change.

## 2. Experimental section

The α-Mn_2_O_3_ prisms were synthesized by our previously reported method [20]. In brief, 8.7 mg of LiMn_2_O_4_ powder and 35 mg of sodium docusate (NaAOT) were dispersed in 15 mL of water using sonication. Then, the pH of the reagent solution was adjusted to 9.0, and then heated in a polytetrafluoroethylene (PTFE) lined stainless steel autoclave for 48 hours at 160°C. The autoclaves were allowed to cool to room temperature over several hours. When the PTFE liner was opened, the solution was a black liquid containing α-Mn_2_O_3_ prisms. Scanning electron microscopy (SEM) images were collected using a Hitachi SU8230 using an acceleration voltage of 3kV. Transmission electron microscopy (TEM) measurements were performed with a JEOL 2100 F microscope using an acceleration voltage of 200kV. TEM images were analyzed using Digital Micrograph. Single-crystal electron diffraction pattern models for the orthorhombic and cubic phase of α-Mn_2_O_3_ were generated with Crystalmaker to index the selected area electron diffraction (SAED) patterns of the prisms. Electron backscattering diffraction measurements were performed on a JEOL JSM-7001 F at 10kV. Temperature-controlled XRD measurements were performed at the SPring-8 BL15XU beamline [21]. The CeO_2_ NIST standard was used as a reference to calibrate the wavelength (λ_xrd_ = 0.653144 Å). Electrode preparation methods including assembly of the disk replaceable glassy carbon electrode (GCE; ALS Co., Ltd. 013362), details of the OER/ORR measurements, and conductivity measurements are provided in the **Supporting Information**.

## 3. Results and discussion

**Figure 1.**
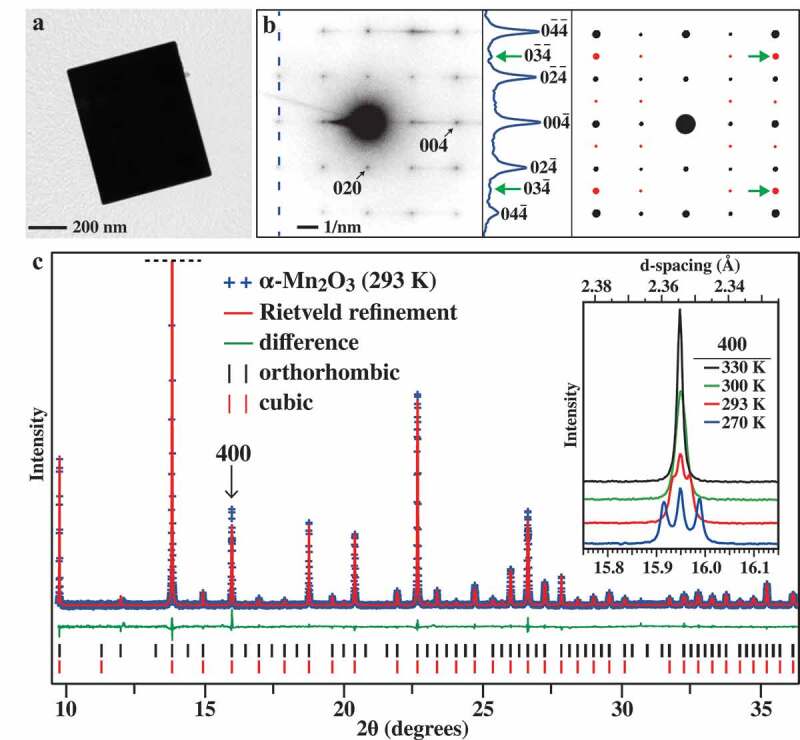
Characterization of the α-Mn_2_O_3_ prisms with TEM and XRD. (**a**) TEM image of a typical prism. (**b; Left**) A SAED pattern collected from a single α-Mn_2_O_3_ prism, which is indexed to the [100] direction. (**b; right**) The single-crystal electron diffraction pattern of the orthorhombic and cubic phase crystals were modeled and projected along the [100] direction. The black reflections are present in both phases. The red reflections are exclusive to the orthorhombic phase. (**b; middle**) A profile scan of the SAED pattern was collected at the blue-dotted line. Most peaks are shared by both phases, but there are two relatively weak peaks at the 03$\overset{ˉ}{4}$ and 0$\overset{ˉ}{3}$$\overset{ˉ}{4}$ reflections that belong to the orthorhombic phase. EBSD measurements show the prisms are bound by 100 facets (see **Fig. S1**). (**c**) A high-resolution X-ray diffraction pattern for α-Mn_2_O_3_ prisms were collected at t = 293 K (λ_xrd_ = 0.653144 Å) and analyzed using Rietveld refinement using the orthorhombic crystal system. The location of the 400 peak in the XRD pattern is indicated with a black arrow at 2θ = 15.95°. (**c; inset**) shows a zoom-in graph of the 400 reflection at various temperatures. As the sample is heated from 270 K to 330 K the degenerate reflections merge as the crystal transforms from orthorhombic to cubic phase

TEM measurements were initially used to characterize the phase of the α-Mn_2_O_3_ prisms (Figure 1(a)). The prisms are relatively thick (∼ 500 nm), but a selected-area electron diffraction (SAED) pattern could be collected and it corresponds to single-crystal α-Mn_2_O_3_ aligned to a ⟨100⟩ crystal direction (Figure 1(b)**; Left**). The cubic and orthorhombic phases of α-Mn_2_O_3_ possess similar reflections and intensities in SAED. Thus, the single-crystal electron diffraction patterns of each phase were modeled using their XRD-derived crystal structures along the [100] direction and superimposed in the rightmost panel of Figure 1(b). The reflections shared by cubic phase and lower-symmetry orthorhombic phase are shown as black circles with their relative intensities illustrated using different diameters (i.e., larger diameters indicate more intense reflections). The red circles indicate reflections that are exclusive to the orthorhombic phase. A line profile was collected from the SAED pattern (see dotted line on Figure 1(b)**; Left**) and plotted as intensity (Figure 1(b)**; Middle**). Numerous refections of the cubic and orthorhombic systems can be observed in the line profile. In addition, we can see a set of weak peaks corresponding to {034} reflections that are only present in

the orthorhombic phase. They indicate that a small amount of orthorhombic phase is present, but the lack of other peaks suggests that the cubic phase is dominant. This observation is unsurprising because of inevitable electron beam heating effects caused by TEM, which makes phase identification challenging in TEM. Regardless, electron microscopy methods are useful for identifying the crystallinity and dominant crystal facets of the materials. Additional SEM images of the particles are shown in **Figure S1A**. Electron backscattering diffraction (EBSD) measurements performed on a single isolated α-Mn_2_O_3_ prism show that the top surface is bound by a {100} facet (**Fig. S1B-E**).

XRD is more frequently used to identify phase changes in materials because the temperature of the sample could be controlled rather trivially to ±1°C at standard pressures using a temperature-control setup. Figure 1(c) shows an XRD pattern of α-Mn_2_O_3_ prisms collected at 293 K (19.85°C). High-resolution XRD patterns obtained at 100 K (−173.15°C), 200 K (−73.15°C), 270 K (−3.15°C), 293 K (19.85°C), 300 K (26.85°C) and 330 K (56.85 C) are located in **Figures S2-S8** and all crystallographic information files (CIFs) are included in the **Supporting Information**. The data were analyzed with the Rietveld refinement method using the bixbyite structures with orthorhombic and cubic cells. Each crystal structure is defined using the abbreviation: Temperature[Crystal System] (e.g., 270 K[Ortho] denotes the 270 K data refined using the orthorhombic system). The simulated curves closely match the high-resolution experimental patterns with low weighted R values (wR < 2%) and exceptional goodness-of-fit (GOF) parameters (**Table S1**). Rietveld analysis shows that the cubic crystal system provides the best numerical match for the α-Mn_2_O_3_ prisms at 300 K. This orthorhombic → cubic transition can be most easily visualized by examining the evolution of the 400 cubic phase peak Figure 1(c)**; inset**. As the sample is cooled below the phase transition temperature it separates into three degenerate 400, 040 and 004 reflection peaks matching the orthorhombic phase (*Pcab* symmetry, space group 61).

**Figure 2.**
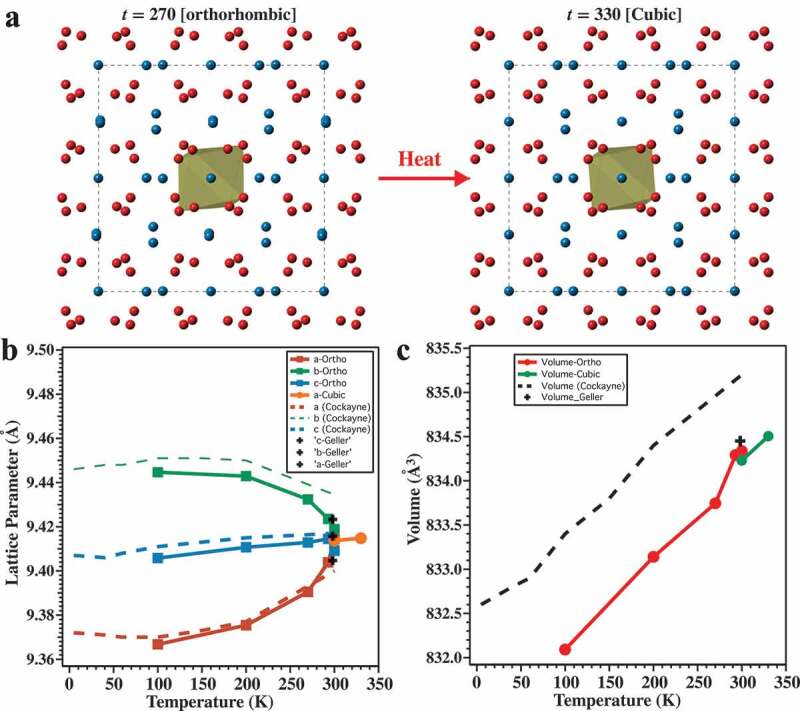
An illustration of orthorhombic (**a; left**) and cubic (**a; right**) phase α-Mn_2_O_3_ at 270 K and 330 K respectively. The crystals are viewed along the [001] axis of the cubic phase. (**b**) A plot of the temperature-dependent lattice parameters for the different crystal systems. (**c**) The unit cell volume versus temperature. The lattice parameters and volumes from [18,22] are included for comparison

Figure 2(a) shows the unit cells of the α-Mn_2_O_3_ structures obtained at 270 K and 330 K. Each structure has Mn^3+^ ions octahedrally coordinated to six O^2-^ ions. The oxygen ions have four Mn neighbors. *Geller* previously explained that the orthorhombic *Pcab* phase of α-Mn_2_O_3_ emerges at low temperatures, caused by Jahn–Teller instabilities that distort the coordination of all 32 Mn atoms in the unit cell [18]. We observed that heating α-Mn_2_O_3_ above the phase transition temperature suppresses or relieves the distortions on 8 out of 32 Mn atoms and the material adopts the cubic phase. Comparisons of our temperature-dependent lattice parameters and unit cell volumes with *Geller* [18] and neutron diffraction data by *Cockayne*, et al. [22] are included in Figure 2(b,c). This data shows that the lattice parameters are relatively continuous up to the phase change >293 K. There appears to be a small discontinuity in the volume change between 293 K[Ortho] → 300 K[Cubic] which might indicate phase transition is higher than first order as predicted earlier [18]. The data shows that the volume change is <0.005% between the 293 K[Ortho] and 300 K[Cubic] unit cell. This indicates that the phase change does not significantly affect the geometric surface area of the catalyst although the ESCA must still be considered and is addressed below.

**Figure 3.**
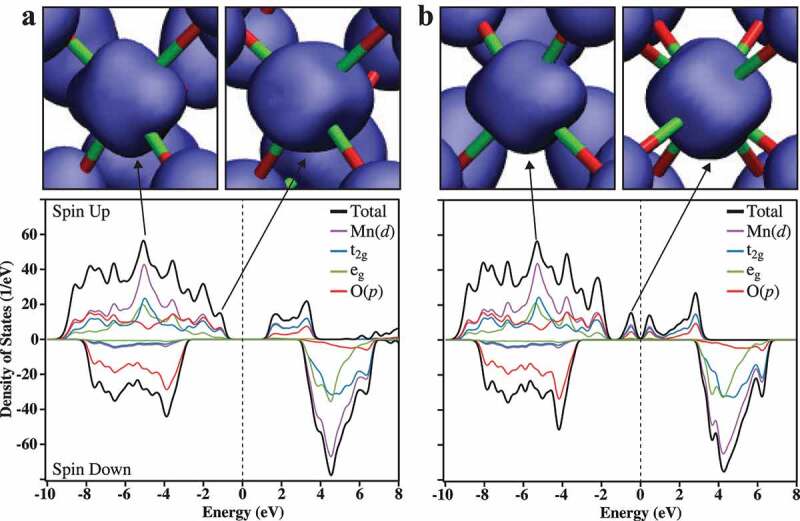
The calculated spin and orbital resolved DOS of 293 K[Ortho] (**a**) and 300 K[Cubic] (**b**). A Lorentzian of width 0.2 eV has been used to broaden the discrete energy levels. The Fermi energy is represented by a vertical dotted line located at 0 eV. In the top region the iso-surfaces (iso-value of 0.4 e/Å^3^) of partial density of states coming from two DOS peaks between −4 to −6 eV, 0 and −2. The atomic bonds are also shown having green (red) spheres representing Mn (O) atoms

Determining how the orthorhombic → cubic phase transformation impacts the electronic band structure and the electrochemical activity of α-Mn_2_O_3_ is the ultimate purpose of these high resolution temperature-resolved XRD measurements. The Rietveld refined crystal structures were input directly into the Vienna Ab initio Simulation Package (VASP) [23,24] implementation of DFT [25] without any geometry optimization and modeled using the PBE0 hybrid functional which is used to reproduce the electronic and magnetic properties of a wide class of materials [26,27]. Previous work showed that hybrid functionals such as PBE0 are able to accurately predict some of the properties of α-Mn_2_O_3_ and show the material is a narrow band gap semiconductor. But these pioneering results are based on low-resolution crystal structures, and electronic properties were calculated using a structure optimized with the HSE functional [28]. We used DFT to calculate the spin and orbital resolved density of states (DOS) of α-Mn_2_O_3_ at *t* = 270 K, 293 K, 300 K and 330 K. The spins of the atoms are dealt with using the fixed spin moment (FSM) method which is a standard technique in DFT to work with magnetic systems. Figure 3(a,b) shows the DOS plots of the 293 K[Ortho] and 300 K[Cubic] structures, with isosurfaces illustrating the charge densities between −4 to −6 eV and −2 to 0 eV. The DOS of the 293 K[Ortho]-derived structure is a narrow band gap semiconductor. Although the 300 K[Cubic] structure had a similar net spin polarization as the 293 K[Ortho] structure, the band gap almost vanishes due to the creation of new electronic states straddling the Fermi level (E_f_). DFT calculations were performed on the 270 K[Ortho] and 330 K[Cubic] structures and they show the same semiconductor to semimetal transition (**Figure S9**).

The large difference in electronegativity between Mn (1.5) and O (3.5) is expected to generate strong ionic bonding. This is demonstrated by plotting the difference charge density iso-surfaces of the 293 K[Ortho] and 300 K[Cubic] structures derived from DFT (**Figure S10**). However, due to the translational symmetry of the 300 K[Cubic] structure, the molecular orbitals are able to transform into Mn 3*d* and O 2*p* bands and mix covalently due to their spatial overlap and energetic similarity [29]. This is shown by plotting the difference charge density iso-surfaces (Figure 3(a,b)**; Top**). These plots reveal the nature of electronic states involved in bonding, for example, in the energy range from −4 to −6 eV there is an overlap of t_2g_ and e_g_ derived states and the corresponding partial charge density iso-surface reflects the coexistence of strongly directional e_g_ (d_z_^2^) states and non-directional t_2g_ states. Whereas in the energy interval between 0 and −2 eV the t_2g_-derived states are predominant and corresponding partial charge density iso-surface well reflects it. There are also differences in the partial charge density iso-surfaces of O atoms, indicating different overlap with neighboring atoms. No significant contributions from 3*d* states in the minority-spin VB close to the E_f_, indicating strong spin polarization in the system. The O 2*s* states are well localized around −25 eV (not shown in Figure 3(a)) and therefore do not contribute significantly to chemical bonding.

The DFT results indicate that the transformation from orthorhombic to cubic phase relieved Jahn–Teller distortions that un-tilted the Mn^3+^O_6_ octahedra and leads to improved Mn 3*d* and O 2*p* covalent bonding and bandwidth. The observed semiconductor-to-semimetal transition can be explained using the well-known Hubbard model [30] considering the fact that PBE0 functional contains 25% of full range Hartree-Fock exchange. In the Hubbard model, electron motion among the atomic sites is controlled by the ratio of intra-atomic Coulomb repulsion strength (U) and 3d bandwidth (W). A large U/W ratio yields electron localization and hence a semiconductor or insulating state, while a small value enhances electron delocalization and a metallic nature. Figure 3 shows that the W value in the cubic phase is nearly 0.5 eV greater compared to the orthorhombic phase, thus interpreting the results using a simplified Hubbard model is relatively convincing.

The electronic states are clearly affected by the phase transformation, but the impact of this change on oxygen electrocatalysis is an open question. Based on a purely ionic point of view, the e_g_ orbitals point directly towards the O atoms and t_2g_ orbitals lie between the O atoms. As a result electrons in the t_2g_ (e_g_) orbitals will be more localized (itinerant) [31]. Figure 3 clearly shows that the phase transformation causes significant changes in the regions close to E_f_. The 293 K[Ortho] phase structure has a majority band gap at 2.2 eV and a minority band gap at 6.1 eV. We mainly consider the majority states since the minority states are far away from E_f_. Note that E_f_ is shifted to 0 eV to simplify the description. The majority-spin VB runs from near −1 to −10 eV and consists of Mn 3*d* and O 2*p* states with a dominant Mn 3*d* character. Moreover, 3*d* states (predominantly t_2g_ states) and 2*p* states overlap in the energy range from −1 to −3 eV and −7 to −9 eV, whereas the Mn 3*d* states dominate the −4 to −6 eV energy range.

The majority-spin conduction band (CB) spans from 1.5 to 4 eV and consists of 3*d* (predominantly t_2g_ states) and 2*p* states. The overlap of 3*d* and 2*p* states gives rise to distinct partial charge density iso-surfaces (spin up plus spin down) as shown in the top region of Figure 3(a). These plots reveal the nature of electronic states involved in bonding, for example, in the energy range from −4 to −6 eV there is an overlap of t_2g_ and e_g_ derived states and the corresponding partial charge density iso-surface reflects the coexistence of strongly directional e_g_ (d_z_^2^) states and non-directional t_2g_ states. Whereas in the energy interval between 0 and −2 eV the t_2g_-derived states are predominant and the corresponding partial charge density iso-surfaces reflect this observation. There are also differences in the partial charge density iso-surfaces of O atoms, indicating different overlap with neighboring atoms. No significant contributions from 3*d* states in the minority-spin VB close to the E_f_, indicating strong spin polarization in the system. The O 2*s* states are well localized around −25 eV (not shown in Figure 3(a)) and therefore do not contribute significantly to chemical bonding.

In the 300 K[Cubic] phase structure in Figure 3(b), states close to E_f_ are dominated by majority states with strongly overlapping Mn 3*d* and O 2*p* states. Again, minority states appear far away from E_f_, indicating a strong spin polarization in the system similar to 293 K[Ortho]. The majority band shows metallic behavior whereas the minority bandgap amounts to 6.5 eV. The majority-spin VB near E_f_ is predominantly an admixture of Mn t_2g_ and O 2*p* states with a minor contribution from Mn e_g_ states. The Mn 3*d* and O 2*p* contributions show similarities in shape close to E_f_ revealing a high degree of covalency in bonding. The O 2*s* states are much lower in energy around −25 eV (not shown in Figure 3(b)) and thus do not participate in the chemical bonding. The overlapping of Mn 3*d* and O 2*p* states gives rise to distinct charge density iso-surfaces as shown in the top region of Figure 3(b). The partial charge density iso-surface in the energy range from −4 to −6 eV depicts a mixed t_2g_ and e_g_ character while predominant t_2g_ character in the energy intervals between 0 to −2 eV and 0 to 2 eV.

In general, improving the bulk conductivity of the electrode improves electrocatalytic performance, but this does not directly address any improvement in the performance of the catalytic active site. Our X-ray diffraction results show that the Mn-O-Mn bond distances become more equivalent in the cubic phase, and DFT simulations suggest this small change in bonding arrangement opens up distinct electronic features close to the E_f_ that have primarily t_2g_ and some e_g_ character. The PBE0 functional we used is known to overestimate band gaps in DFT [27], so there may be more of these low-lying electronic states that provide a lower energy barrier to enable the coexistence of Mn oxidation states that is critical for catalytic activity.

To investigate how the temperature-dependent phase transition directly affects oxygen electrocatalysis, we performed OER and ORR catalysis experiments in alkaline media at different temperatures. The α-Mn_2_O_3_ prisms were heated in air at 480°C on the removable GCE and the electrode was then assembled. The details of the electrode preparation method are provided in the **Supporting Information**. Linear sweep voltammetry (LSV) measurements were performed in O_2_-saturated 0.1 M KOH and scanned using a rotating disc electrode (RDE) at 1600 rpm in a three-electrode electrochemical cell with temperature controlled via a recirculating water bath setup (EYELA NTT-20S; ±0.05 C). Initially, we compared the α-Mn_2_O_3_ prism electrodes that had been heat-treated versus electrodes where the step had been omitted, performing LSVs at a temperature of 25°C to replicate our earlier work performed at room temperature (**Figure S11**) [20]. Again, the heat treated prisms exhibited superior performance for both OER/ORR at 25°C, and even better performance at 50°C. **Table S2** briefly summarizes the performance of these electrodes versus commercial α-Mn_2_O_3_, Pt/C and RuO_2_. Next, the electrochemical properties of the heat-treated α-Mn_2_O_3_ prisms were measured stepwise at temperatures from *t* = 15°C to 50°C (i.e. 288 K to 323 K). LSV curves and Tafel plots for the α-Mn_2_O_3_ prisms were collected and plotted in **Figure S12**. The OER/ORR Tafel slopes in addition to the ORR potentials at −3 mA⋅cm^−2^ and OER potentials at 10 mA⋅cm^−2^ at various temperatures are summarized in **Table S3**. The onset potentials at various temperatures were plotted in **Figure S13**, and there is a jump in performance near the phase transition temperature for both ORR and OER. These results show that the α-Mn_2_O_3_ prisms have higher activity at a higher temperature. Temperature will have only negligible affect on the pH of the 0.1 M KOH electrolyte because it has a strong dissociation constant. However, higher temperatures will typically increase the kinetics of bond cleavages and rearrangements, thus a closer look for change around the orthorhombic → cubic phase transition temperature is necessary. Additionally, we carried out conductivity measurements on the α-Mn_2_O_3_ powder and found no apparent shift in the conductivity over 4 cycles from 0°C to 50°C (**Figure S14**). This observation further confirms that the electrochemical active surface area (ECSA) of the powder is constant and is not affected by the phase change.

**Figure 4.**
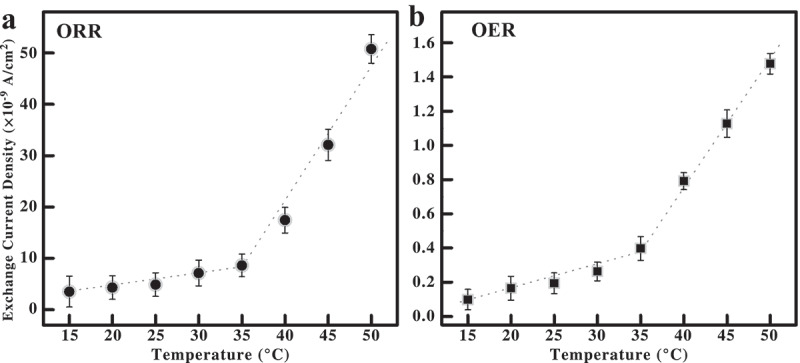
Exchange current density for α-Mn_2_O_3_ electrodes at different temperatures obtained using the Tafel plots of the (**a**) ORR and (**b**) OER regions

The exchange current density (i_0_) reflects the intrinsic rate of electron transfer versus area in addition to the number of active sites and their quality. Our XRD and conductivity measurements (Figure 2(c)**, S14**) show that any change in the surface area of α-Mn_2_O_3_ with temperature is negligible. Thus, if the same electrode is used in the temperature-controlled electrochemical experiments over a modest range, we reasoned that the surface area of the electrode should be relatively constant, so i_0_ will provide information on the quality of the active site. Figure 4 shows i_0_ versus temperature in the ORR (**a**) and OER (**b**) regions of the Tafel plots. In both ORR and OER we observed a marked increase and inflection in i_0_ near the phase transition of α-Mn_2_O_3_, indicating the cubic phase has better active sites. The temperature-dependent conductivity of neat 0.1 M KOH by itself is linear and increases at a negligible rate in this temperature range (∼0.2 mS/cm/°C) so this change in i_0_ is not due to the electrolyte [32]. The inflection in i_0_ is ∼35°C slightly higher than the phase transition temperature of the dry α-Mn_2_O_3_ powder in XRD measurements. However, it is known that the phase of manganese oxides is highly sensitive to oxygen partial pressure, hydration and formation of surface passivating layers [33,34]. We attribute this small discrepancy in i_0_ to the high concentration of oxygen and alkalinity of the electrolyte, which modifies the surface passivation of the material and likely increases the orthorhombic → cubic phase transition temperature.

## 4. Conclusions

We used phase pure α-Mn_2_O_3_ prisms to obtain the highest resolution XRD patterns to date for various temperatures around the orthorhombic → cubic phase transition. This near room temperature phase change causes a subtle rearrangement in the bonding of α-Mn_2_O_3_ and demonstrates the suppression of Jahn–Teller distortions above the phase transition temperature. The crystal structures were input into DFT, and indicate that the orthorhombic → cubic phase transition causes the material to transform from semiconducting to semi-metallic and opens up states around the E_f_, in addition to improving the covalency of the Mn-O-Mn bonds. The OER and ORR performance of the α-Mn_2_O_3_ prisms were examined in a temperature-controlled electrocatalytic setup using the same electrode to eliminate contributions from different electrode preparation methods. These results show that the activity of the catalyst improves at higher temperatures in an aqueous alkaline environment. A closer examination of the i_0_ shows the electrode has lower activation polarization losses and better performance above the phase transition temperature. The combined structural, theoretical and electrochemical measurements here demonstrate how a tiny change in crystal structure modifies the electronic properties of a manganese oxide material and impacts its catalytic properties. Manganese oxides are inexpensive catalysts for oxygen electrochemistry because they are abundant in the Earth’s crust, and even thought to be present in high concentrations in fracture zones on the surface of Mars [35]. Overall this work suggests that the phase behavior of manganese oxides should be considered when analyzing their electronic and catalytic properties, and indicates that there is much to learn about the phase-dependent electrocatalytic properties of binary and mixed metal oxides.

## Supplementary Material

Supplemental Material

Supplemental Material

## Supporting information description

Powder XRD crystal structures for 100 K[Ortho], 200 K[Ortho], 270 K[Ortho], 293 K[Ortho], 300 K[Ortho], 300 K[Cubic] and 330 K[Cubic] are included as crystallographic information files. The supporting information (SI) document contains graphs showing the experimental data and Rietveld refinement, as well as the reflections of the orthorhombic and cubic phases, and goodness-of-fit (GOF) and R_w_ parameters. It also contains the DOS of 270 K[Ortho] and 330 K[Cubic] derived from DFT, as well as the iso-surfaces of the 293 K[Ortho] and 300 K[Cubic] structures. The electrochemical data including LSVs and Tafel plots for OER/ORR are also included, with tables summarizing performance at various temperatures. Finally, details of electrode preparation and the electrochemical measurements are enclosed.
