# Supplementary material for: Temperature-dependent electronic structure of bixbyite α-Mn2O3 and the importance of a subtle structural change on oxygen electrocatalysis
Source: Sci Technol Adv Mater. 2021 Apr 9;22(1):141–9. doi: 10.1080/14686996.2020.1868949 (PMC8043564; doi:10.1080/14686996.2020.1868949)
Supplement: Supplemental Material [file TSTA_A_1868949_SM9178.pdf]

Temperature-dependent electronic structure of bixbyite *α-Mn_2O_3* and the importance of a subtle structural change on oxygen electrocatalysis

Junais Mokkath,[a,b,‡] *Maryam Jahan,*[a,‡] *Masahiko Tanaka,*[c] *Satoshi Tominaka,*[a,*] *Joel Henzie* [a,*]

1. International Center for Materials Architectonics (MANA), National Institute for Materials Science (NIMS), 1-1 Namiki, Tsukuba, Ibaraki, 305-0044 Japan

2. Department of Physics, Kuwait College of Science And Technology, Doha Area, 7th Ring Road, P.O. Box 27235, Kuwait

2. Synchrotron X-ray Station at SPring-8, National Institute for Materials Science, Kouto 1-1-1, Sayo, Hyogo 679-5148, Japan

Contents:

I. Supporting Figures and Tables

Figure S1. *SEM image and EBSD analysis showing that the surface of the* α-*Mn_2O_3 prisms are bound by {100} facets.*

Figure S2. *A high-resolution X-ray diffraction pattern for* α-*Mn_2O_3 performed at t = 100 K (λ = 0.653144A). Rietveld refinement was performed using an orthorhombic crystal system.*

Figure S3. *A high-resolution X-ray diffraction pattern for* α-Mn_2O_3 *performed at t = 200 K (λ = 0.653144A). Rietveld refinement was performed using an orthorhombic crystal system.*

Figure S4. *A high-resolution X-ray diffraction pattern for* α-Mn_2O_3 *performed at t = 270 K (λ = 0.653144A). Rietveld refinement was performed using an orthorhombic crystal system.*

Figure S5. *A high-resolution X-ray diffraction pattern for* α-Mn_2O_3 *performed at t = 293 K (λ = 0.653144A). Rietveld refinement was performed using an orthorhombic crystal system.*

Figure S6. *A high-resolution X-ray diffraction pattern for* α-Mn_2O_3 *performed at t = 300 K (λ = 0.653144A). Rietveld refinement was performed using an orthorhombic crystal system.*

Figure S7. *A high-resolution X-ray diffraction pattern for* α-Mn_2O_3 *performed at t = 300 K (λ = 0.653144A). Rietveld refinement was performed using an cubic crystal system.*

Figure S8. *A high-resolution X-ray diffraction pattern for* α-Mn_2O_3 *performed at t = 330 K (λ = 0.653144A). Rietveld refinement was performed using an cubic crystal system.*

Figure S9. *The calculated spin and orbital resolved DOS of orthorhombic phase @270K (left) and cubic phase @330K.*

Figure S10. *Isosurfaces showing the difference in charge density between the 293K@Orthorhombic phase and 300K@Cubic phase.*

Figure S11. *LSVs showing the OER/ORR performance of the as-prepared α-Mn_2O_3 prisms that were simply dropcast on the GCE electode and measured at 25°C, α-Mn_2O_3 prisms that had been heated to 480°C for 10 hours then cooled and measured at 25*°C*, α-Mn_2O_3 prisms that had been heated to 480°C for 10 hours then cooled and measured at 50*°C, *Pt/C and RuO_2.*

Figure S12. *LSVs and tafel plots of the α-Mn_2O_3 prisms in ORR and OER regions scanned in O_2-saturated 0.1 M KOH at various temperatures; 15, 20, 25, 30, 35, 40, 45, 50*°C *at 1600 rpm.*

Figure S13. *Temperature-dependent onset potentials for ORR and OER.*

Figure S14. *Temperature-dependent conductivity measurements performed on a powder composed of Mn_2O_3 prisms.*

Table S1. *Unit cells, Goodness-of-fits (GOF) and weighted R-factor (wR) parameters for the Rietveld refinements of the different crystal systems used to fit the α-Mn_2O_3 structures.*

Table S2. *Comparison of oxygen electrode activities of α-Mn_2O_3 prisms different thermal treatment methods and different conditions. The catalytic activities of commercial Pt/C and commercial α-Mn_2O_3 powder.*

Table S3. *A summary of the OER/ORR performance of the materials in **Figure S12**.*

II. Crystal structure data used in DFT calculations

III. Description of the electrode preparation, conductivity measurements, and electrochemical measurements.

IV. References

I. Supporting Figures and Tables

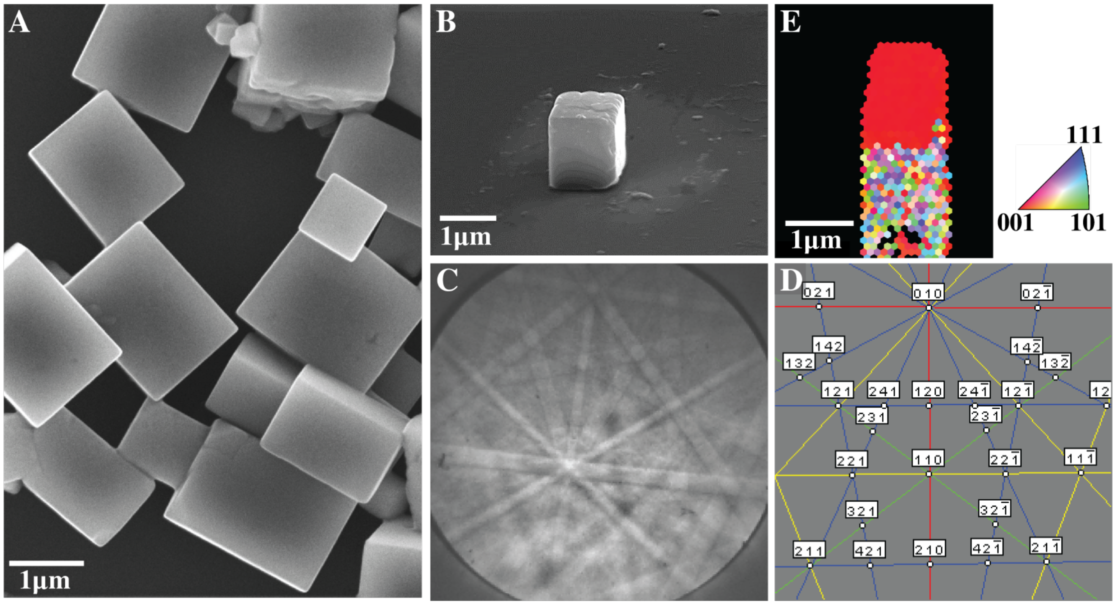

Figure S1. *(**A**) SEM image of the* α*-*Mn_2O_3 *prisms after hydrothermal synthesis using the cation bridging method. Electron backscattering diffraction (EBSD) measurements were performed on a single prism (**B**) to further prove the crystallinity and native crystal habit. (**C**) The prism was tilted toward the EBSD detector to generate a Kikuchi pattern. The Kikuchi lines were indexed to identify the phase and crystal orientation (D). (E) shows the inverse pole figure map indicating that the top surface of the the* α*-*Mn_2O_3 *prism is bound by a {001} facet, confirming our observations using high-resolution TEM.*

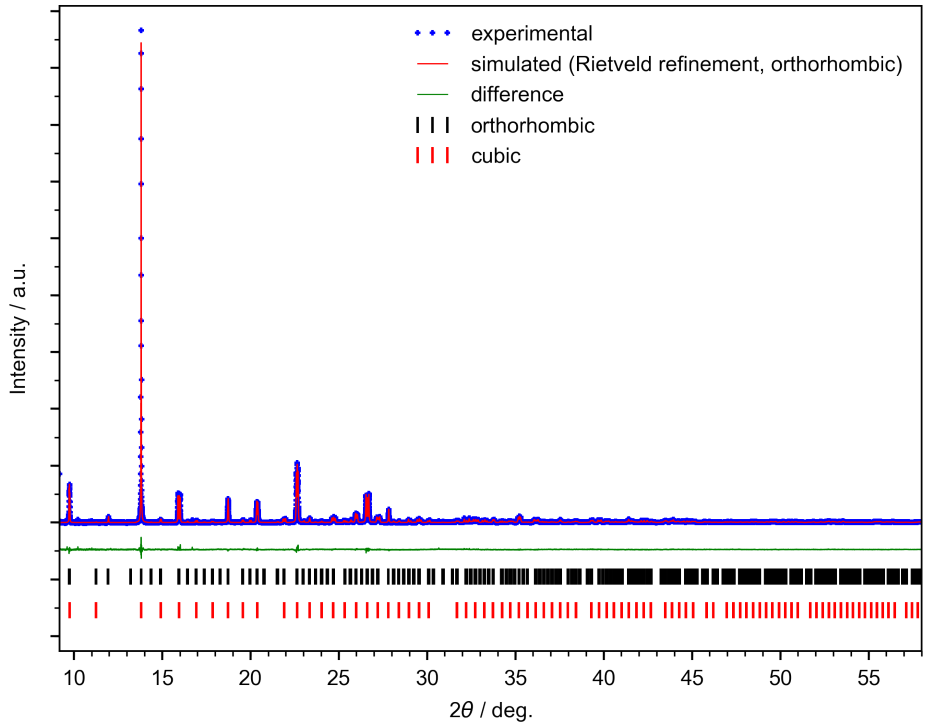

Figure S2. *A high-resolution X-ray diffraction pattern for* α*-*Mn_2O_3 *performed at* ***t = 100 K*** *showing both experimental data and Rietveld refinement using the* ***orthorhomic phase*** *(*λ_{XRD} *= 0.653144 Å). Bragg reflections for orthorhombic and cubic phase* α*-*Mn_2O_3 *are at the bottom for reference.*

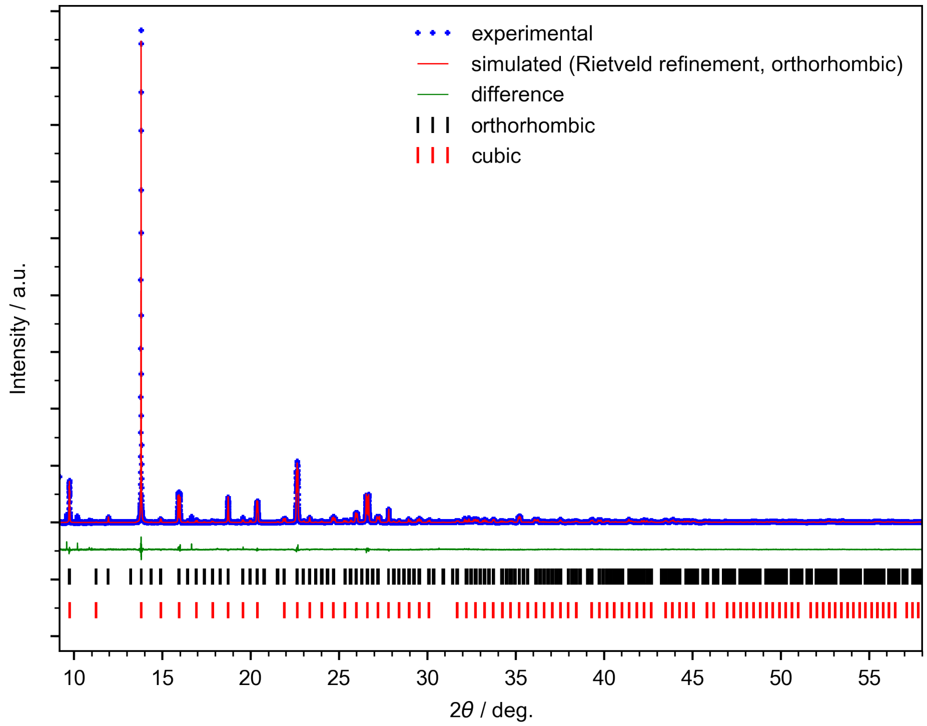

Figure S3. *A high-resolution X-ray diffraction pattern for* α*-Mn_2O_3 performed at* ***t = 200 K*** *showing both experimental data and Rietveld refinement using the* ***orthorhomic phase*** *(λ_{XRD} = 0.653144 Å). Bragg reflections for orthorhombic and cubic phase* α*-Mn_2O_3 are at the bottom for reference.*

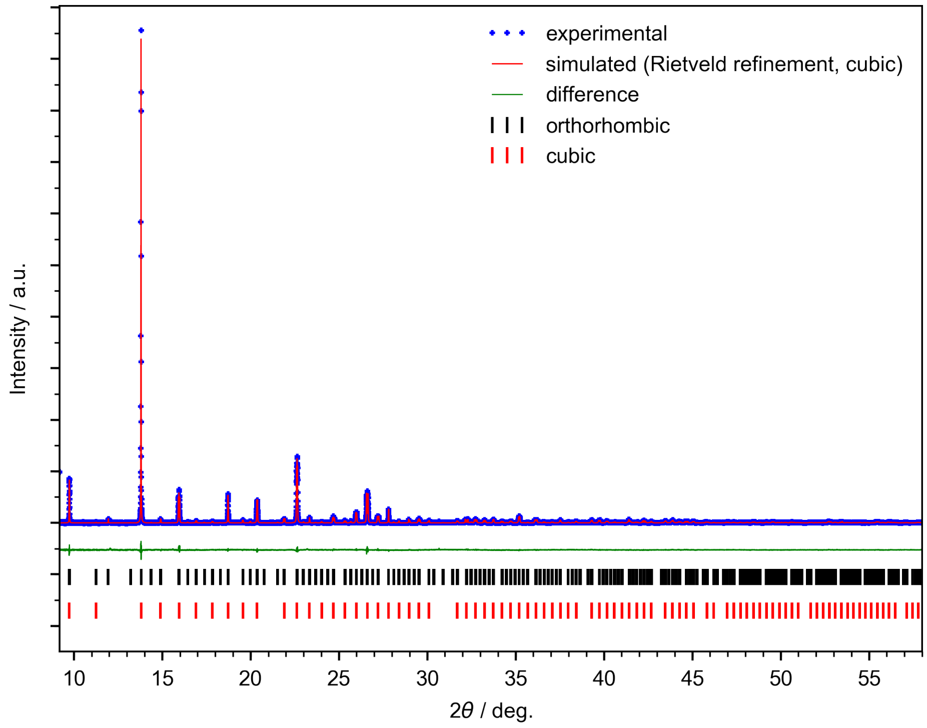

Figure S4. *A high-resolution X-ray diffraction pattern for* α*-Mn_2O_3 performed at* ***t = 270 K*** *showing both experimental data and Rietveld refinement using the* ***orthorhombic phase*** *(λ_{XRD} = 0.653144 Å). Bragg reflections for orthorhombic and cubic phase* α*-Mn_2O_3 are at the bottom for reference.*

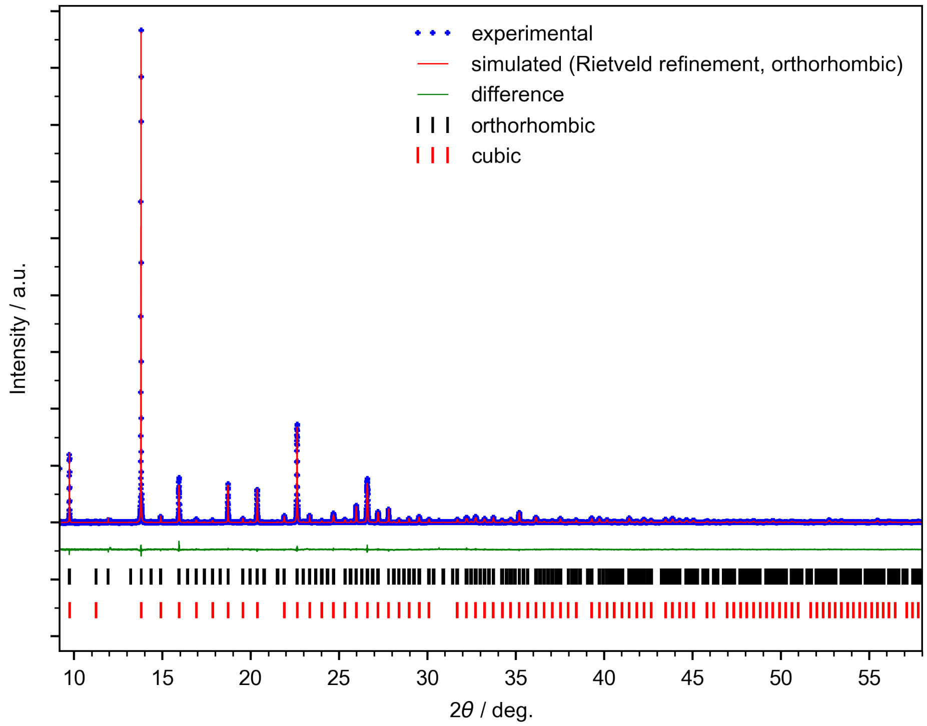

Figure S5. *A high-resolution X-ray diffraction pattern for* α-Mn_2O_3 *performed at* ***t = 293 K*** *showing both experimental data and Rietveld refinement using the* ***orthorhombic phase*** *(*λ_{XRD} *= 0.653144 Å). Bragg reflections for orthorhombic and cubic phase* α-Mn_2O_3 *are at the bottom for reference.*

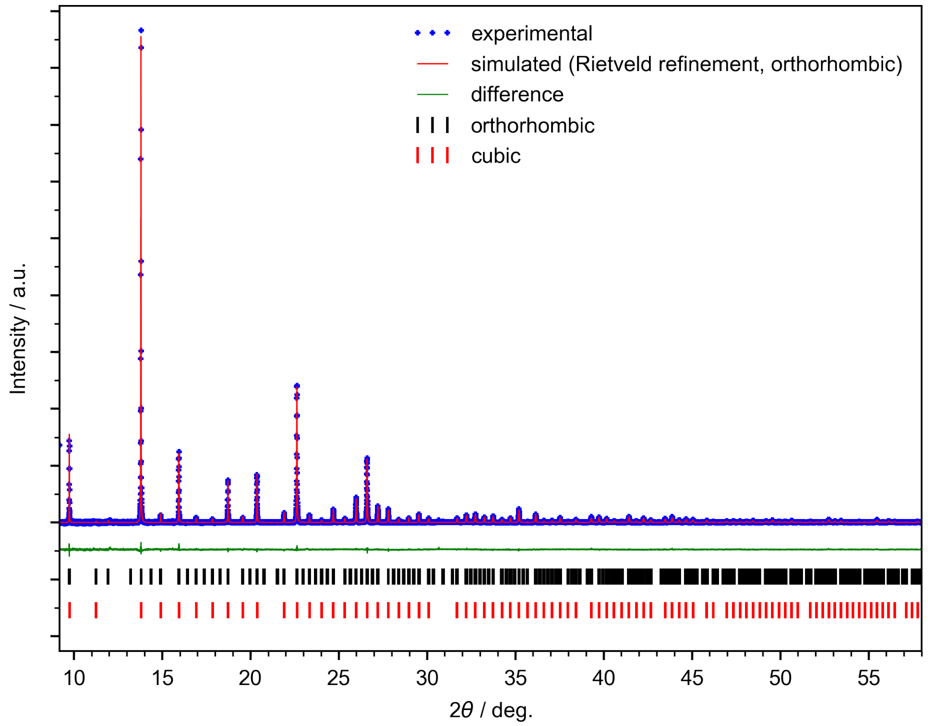

Figure S6. *A high-resolution X-ray diffraction pattern for* α-*Mn_2O_3 performed at* ***t = 300 K*** *showing both experimental data and Rietveld refinement using the* ***orthorhombic phase*** *(λ_{XRD} = 0.653144 Å). Bragg reflections for orthorhombic and cubic phase* α-*Mn_2O_3 are at the bottom for reference.*

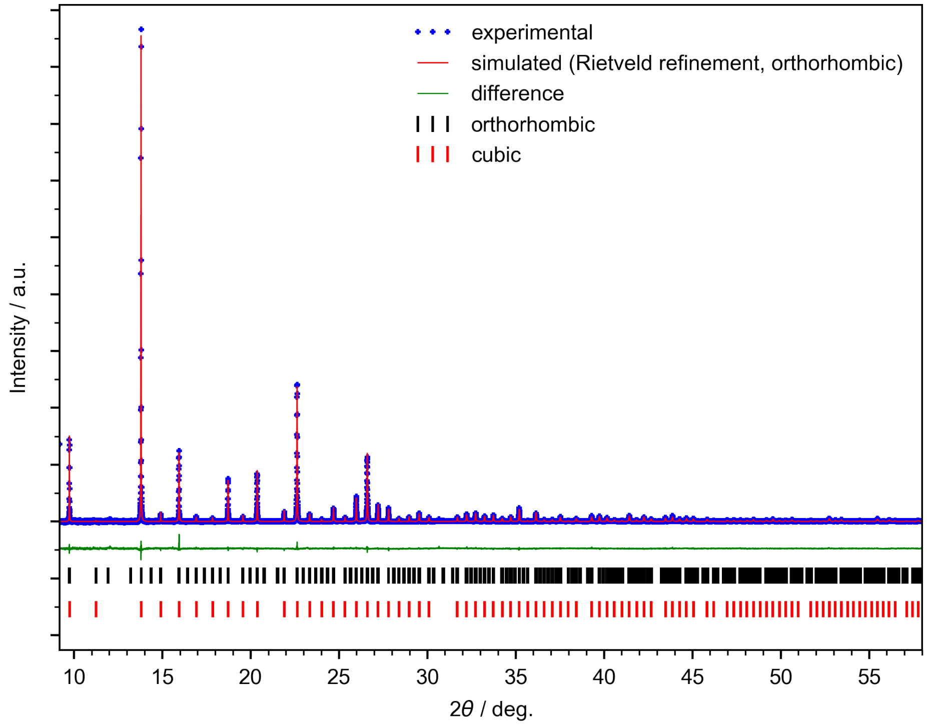

Figure S7. *A high-resolution X-ray diffraction pattern for α-Mn_2O_3 performed at* ***t = 300 K*** *showing both experimental data and Rietveld refinement using the* ***cubic phase*** *(λ_{XRD} = 0.653144 Å). Bragg reflections for orthorhombic and cubic phase α-Mn_2O_3 are at the bottom for reference.*

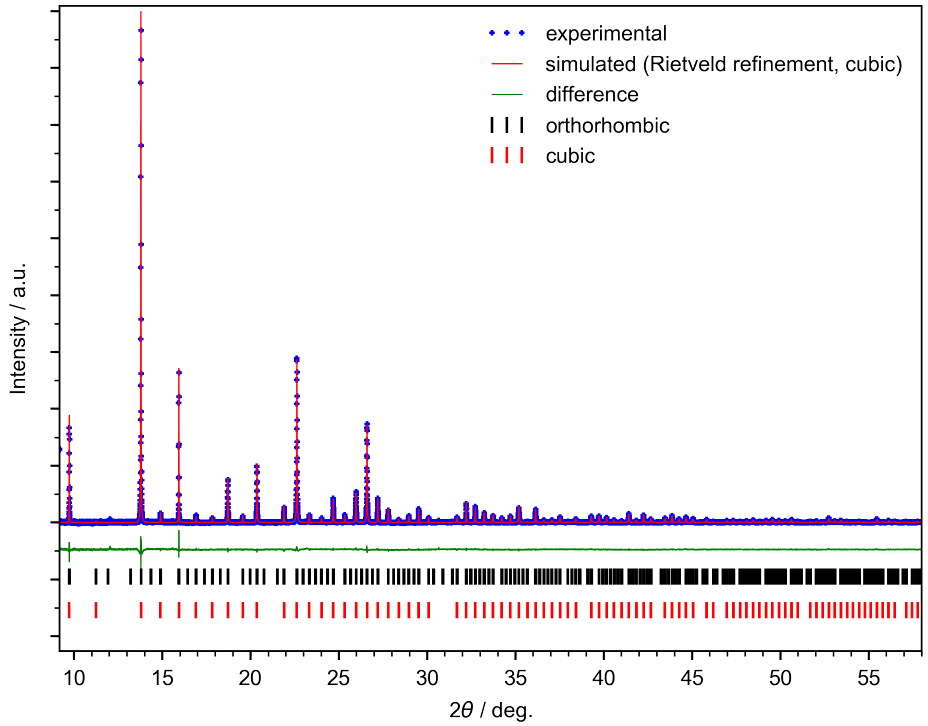

Figure S8. *A high-resolution X-ray diffraction pattern for* α*-*Mn_2O_3 *performed at* ***t = 330 K*** *showing both experimental data and Rietveld refinement using the* ***cubic phase*** *(*λ_{XRD} *= 0.653144 Å). Bragg reflections for orthorhombic and cubic phase* α*-*Mn_2O_3 *are at the bottom for reference.*

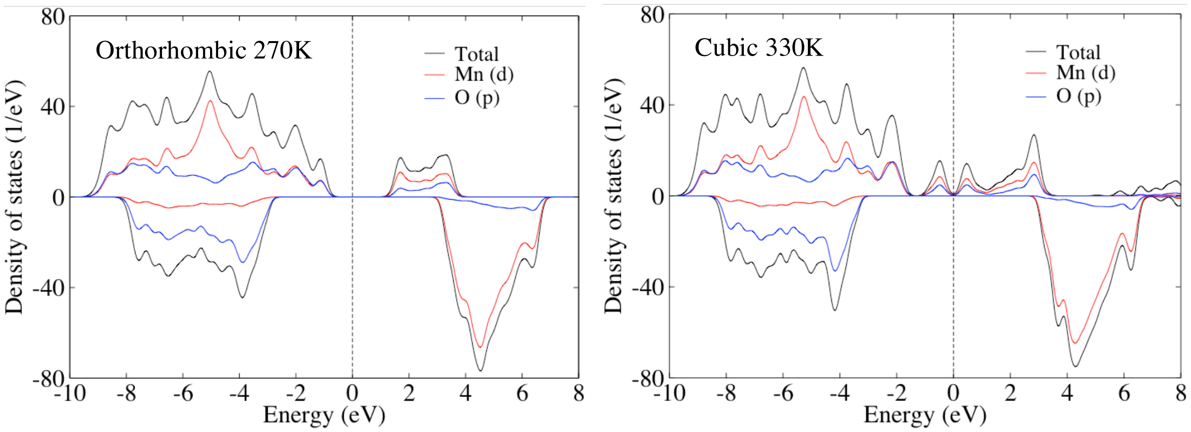

Figure S9. *The calculated spin and orbital resolved DOS of orthorhombic phase @270K (left) and cubic phase @330K. A Lorentzian of width 0.2 eV has been used to broaden the discrete energy levels. The Fermi energy is represented with a vertical broken line located at 0 eV.*

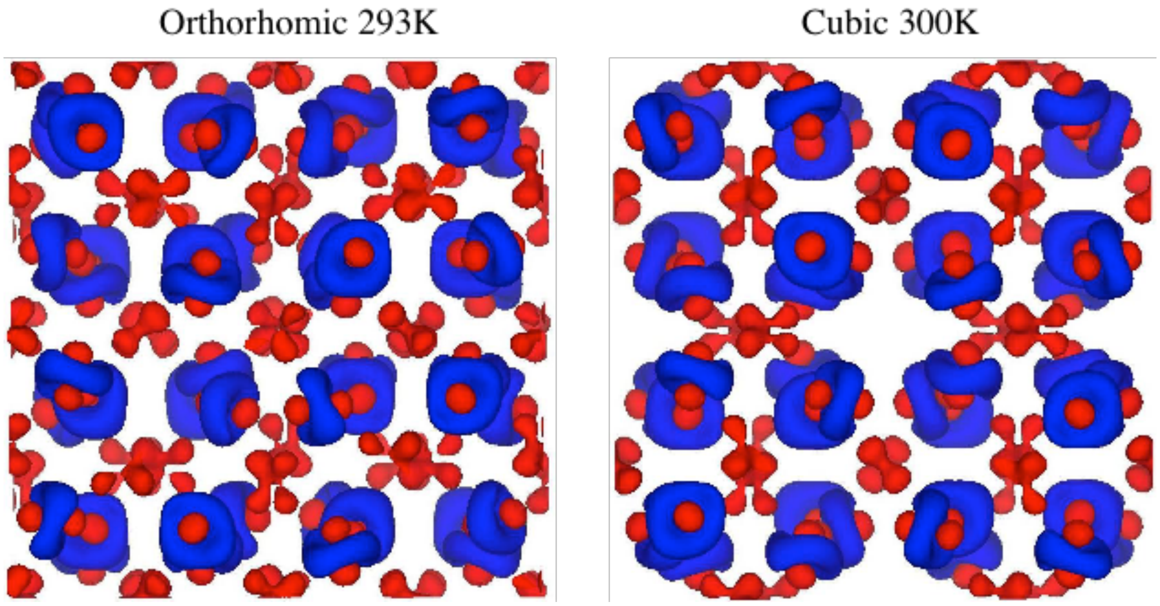

Figure S10. *Isosurfaces showing the difference in charge density between the unit cells of the 293K@Orthorhombic phase **(left)** and 300K@Cubic phase **(right)** structures. The blue (red) color indicates electron accumulation (depletion) regions, respectively. The isovalue is 0.01 electron/Å^3. The unit cells of each structure is roughly ~9.4Å. The specific parameters of the unit cells are described in **Table S1**.*

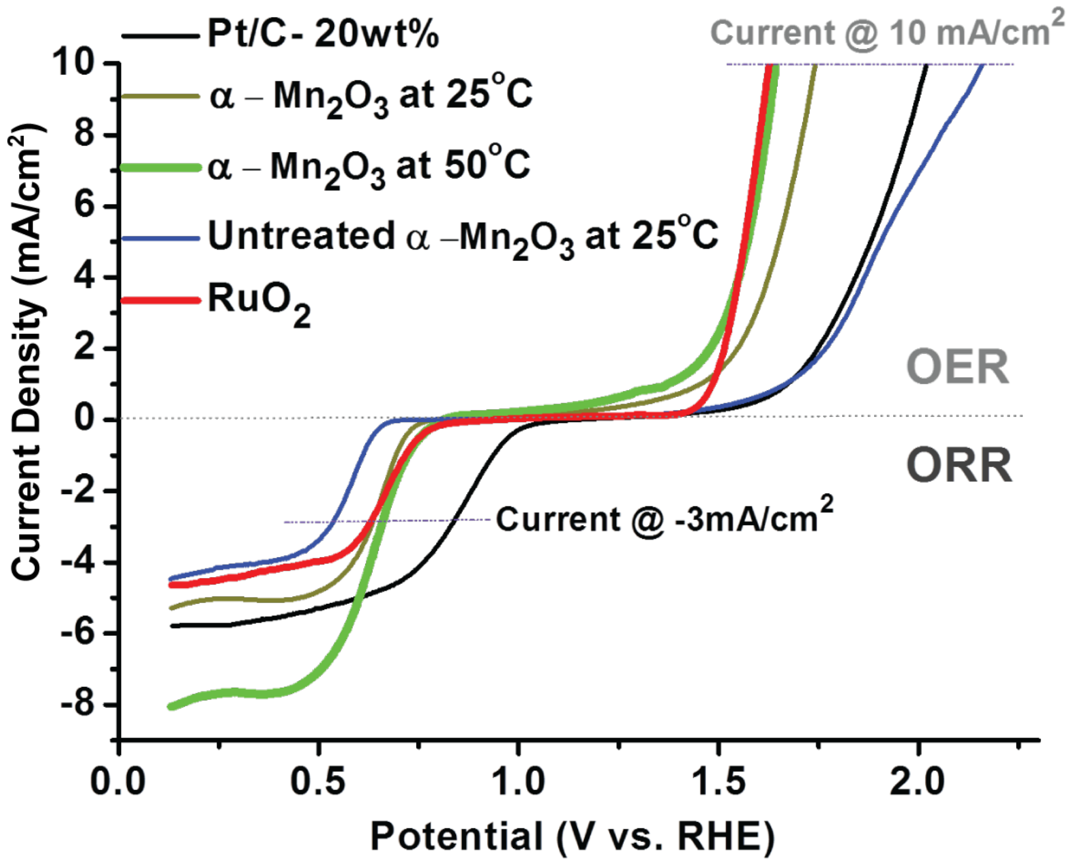

Figure S11. *LSVs showing the OER/ORR performance of the as-prepared α-Mn_2O_3 prisms that were dropcast on the GCE electode and measured at 25°C (**blue curve**), α-Mn_2O_3 prisms that had been heated to 480°C for 10 hours then cooled and measured at 25*°C *(**dark green curve**), α-Mn_2O_3 prisms that had been heated to 480°C for 10 hours then cooled and measured at 50*°C *C (**bright green curve**), Pt/C 20 wt% C (**black curve**) and RuO_2 (**red curve**).*

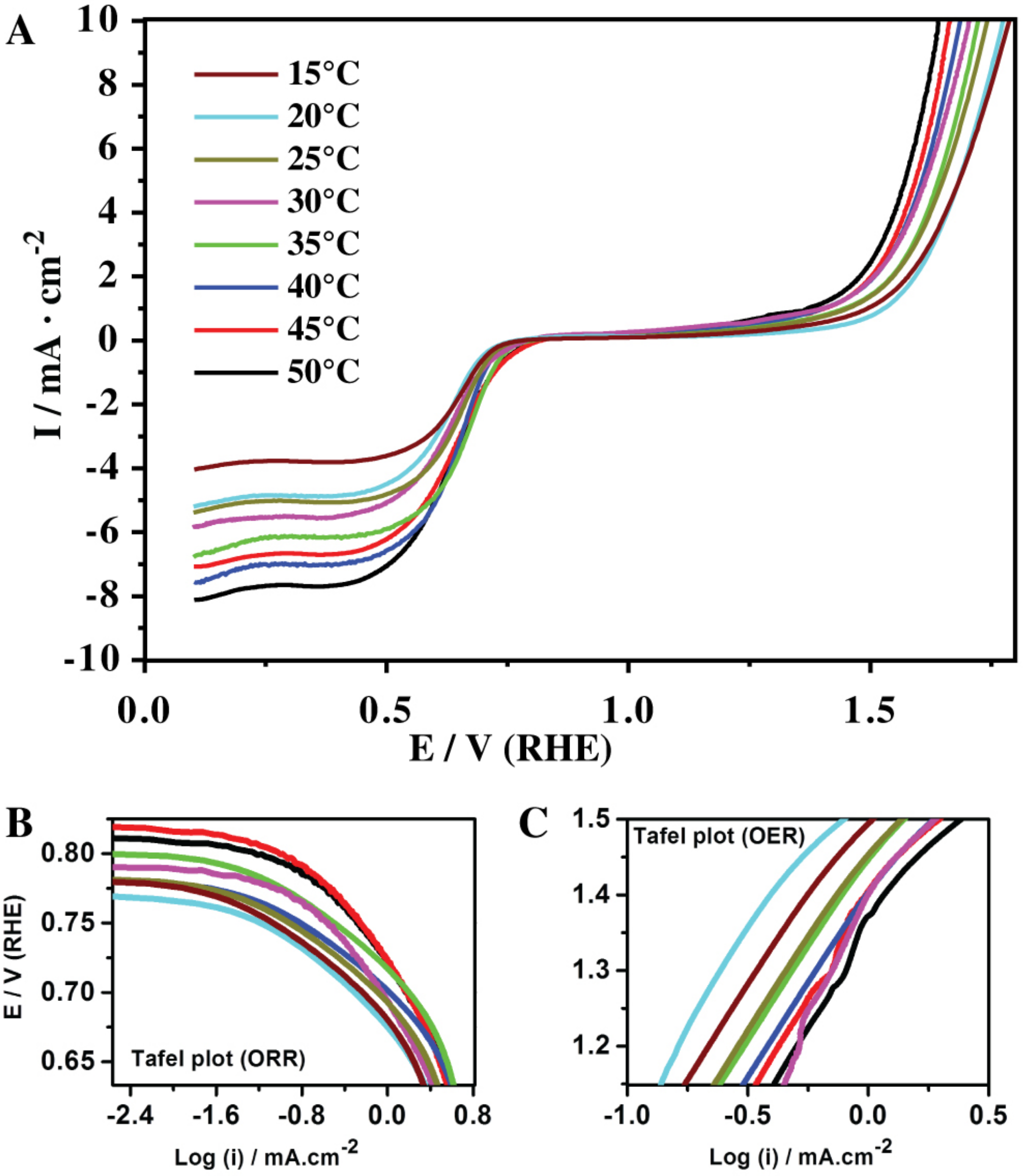

Figure S12. **(A)** *LSV of heated α-Mn_2O_3 in ORR and OER regions scanned in O_2-saturated 0.1 M KOH at various temperatures; 15, 20, 25, 30, 35, 40, 45, 50*°C *at 1600 rpm.* **(B)** *Tafel plots for the ORR region.* **(C)** *Tafel plots for the OER region.*

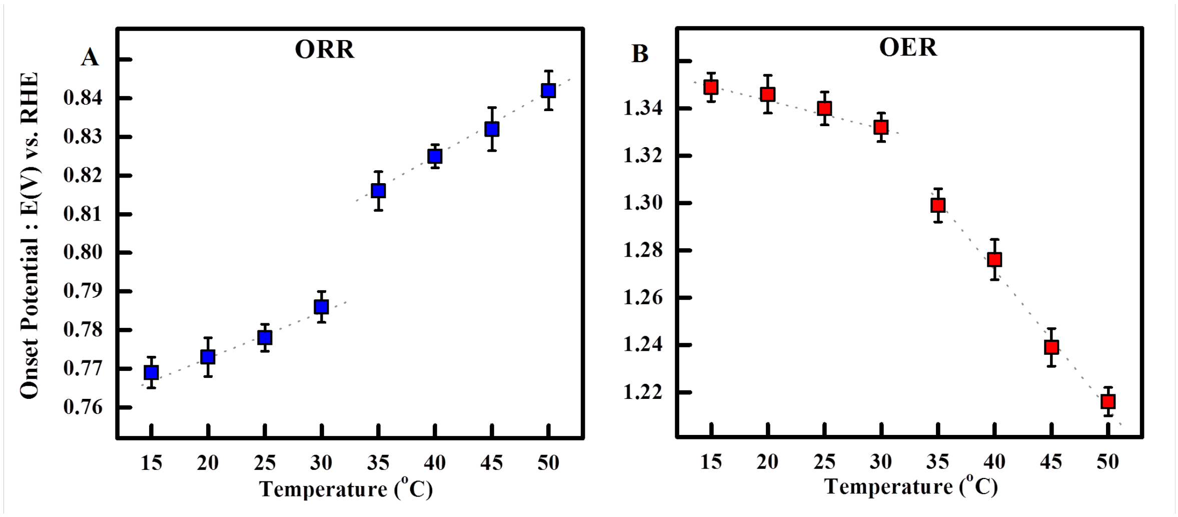

Figure S13. *Temperature-dependent onset potentials for ORR* (**A**) *and OER* (**B**) *obtained in O_2-saturated 0.1 M KOH at various temperatures at 1600 rpm.*

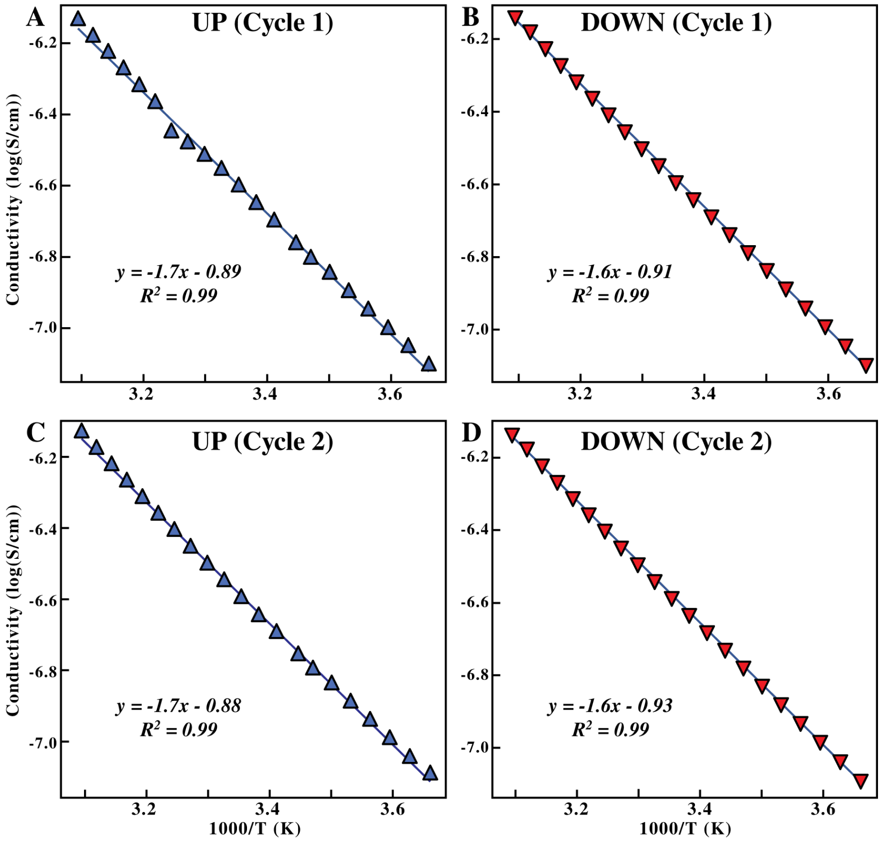

Figure S14. *Temperature-dependent conductivity measurements performed on a powder composed of Mn_2O_3 prisms.*

Temp (K)	Crystal System	*(in Angstroms)* *a*	*b*	*c*	Goodness-of-Fit	weighted R-factor (wR)
330	Cubic	9.41478(2)			1.82	2.01%
300	Cubic	9.41374(3)			1.64	1.48%
300	Ortho	9.41436(5)	9.41890(6)	9.40916(4)	1.49	1.35%
293	Ortho	9.40387(6)	9.42354(6)	9.41450(6)	1.60	1.77%
270	Ortho	9.39051(8)	9.43238(8)	9.41289(8)	1.64	1.81%
200	Ortho	9.37539(10)	9.44292(10)	9.41071(10)	1.97	2.10%
100	Ortho	9.36674(9)	9.44466(9)	9.40579(9)	1.87	2.08%

Table S1. *Unit cells, Goodness-of-fits (GOF) and weighted R-factor (wR) parameters for the Rietveld refinements of the different crystal systems used to fit the* α-Mn_2O_3 *structures.*

Table S2. Variation of OER/ORR performance of thermally-treated for α-Mn_2O_3 prism electrodes using LSVs performed at various temperatures.

Temp (°C)		Tafel slope High overpotential mV/decade	ORR:E(V) at I = -3 mA.cm^{-2}	OER:E(V) at I = 10 mA.cm^{-2}	Oxygen electrode activity Δ(OER-ORR): E(V) vs. RHE (*Smaller is better*)
Pt/C 20wt% (commercial)	ORR	121	0.83	2.01	1.18
	OER	120			
RuO_2 (commercial)	ORR	125	0.63	1.62	0.99
	OER	118			
Unheated α-Mn_2O_3 (at 25 °C)	ORR	135	0.52	2.16	1.64
	OER	154			
480°C heat-treated α-Mn_2O_3 (at 25 °C)	ORR	128	0.63	1.74	1.11
	OER	146			
α-Mn_2O_3 (at 50 °C) [Cubic Phase]	ORR	163	0.66	1.63	0.97
	OER	177			
commercial Mn_2O_3 (at 25 °C)	ORR	117	0.14	2.24	2.10
	OER	297			
Heated commercial Mn_2O_3 (at 25 °C)	ORR	135	0.40	2.39	1.99
	OER	266			
	OER	147			

Table S3. Variation of OER/ORR performance of thermally-treated for α-Mn_2O_3 prism electrodes using LSVs performed at various temperatures.

Temp (^oC)		Tafel slope High overpotential mV/decade	ORR:E(V) at I = -3 mA.cm^{-2}	OER:E(V) at I = 10 mA.cm^{-2}	Oxygen electrode activity Δ(OER-ORR): E(V) vs. RHE (*Smaller is better*)
**50**	**ORR**	**163**	**0.655**	**1.630**	**0.975**
	**OER**	**177**			
**45**	**ORR**	**158**	**0.651**	**1.663**	**1.012**
	**OER**	**173**			
**40**	**ORR**	**145**	**0.653**	**1.686**	**1.033**
	**OER**	**167**			
**35**	**ORR**	**139**	**0.665**	**1.704**	**1.039**
	**OER**	**162**			
**30**	**ORR**	**136**	**0.621**	**1.723**	**1.102**
	**OER**	**155**			
**25**	**ORR**	**128**	**0.630**	**1.741**	**1.111**
	**OER**	**146**			
**20**	**ORR**	**123**	**0.602**	**1.774**	**1.172**
	**OER**	**143**			
**15**	**ORR**	**121**	**0.584**	**1.787**	**1.203**
	**OER**	**147**			

II. Crystal structure data used in DFT calculations

Orthorhombic @273K

```
# phase info for Mn2 O3-ortho follows
_pd_phase_name  "Mn2 O3-ortho"
_cell_length_a  9.39051
_cell_length_b  9.43238
_cell_length_c  9.412891
_cell_angle_alpha  90
_cell_angle_beta   90
_cell_angle_gamma  90
_cell_volume  833.745
_symmetry_cell_setting  orthorhombic
_symmetry_space_group_name_H-M  "P c a b"
loop_
   _space_group_symop_id
   _space_group_symop_operation_xyz
    1  x,y,z
    2  1/2-x,y,1/2+z
    3  1/2+x,1/2-y,z
    4  -x,1/2-y,1/2+z
    5  -x,-y,-z
    6  1/2+x,-y,1/2-z
    7  1/2-x,1/2+y,-z
    8  x,1/2+y,1/2-z

# ATOMIC COORDINATES AND DISPLACEMENT PARAMETERS
loop_
   _atom_site_label
   _atom_site_type_symbol
   _atom_site_fract_x
   _atom_site_fract_y
   _atom_site_fract_z
   _atom_site_occupancy
   _atom_site_adp_type
   _atom_site_U_iso_or_equiv
   _atom_site_symmetry_multiplicity
Mn1   Mn3+ 0.00000      0.00000      0.00000      1.000   Uiso 0.00481(13) 4
Mn2   Mn3+ 0.00000      0.00000      0.50000      1.000   Uiso 0.00481(13) 4
Mn3   Mn3+ 0.28383(10)  0.25621(30)  -0.00845(27) 1.000   Uiso 0.00481(13) 8
Mn4   Mn3+ 0.00790(29)  0.28503(10)  0.24384(28) 1.000    Uiso 0.00481(13) 8
Mn5   Mn3+ 0.2540(3)    0.0012(4)    0.28584(11) 1.000    Uiso 0.00481(13) 8
O1    O2-  0.1377(10)  -0.0868(12)  0.1598(9)    1.000    Uiso 0.0080(4)  8
O2    O2-  0.1453(11)  0.1289(14)  -0.0745(13)   1.000    Uiso 0.0080(4)  8
O3    O2-  -0.0794(10)  0.1459(12)  0.1206(9)    1.000    Uiso 0.0080(4)  8
O4    O2-  -0.3777(11)  0.4141(11)  -0.3562(10)  1.000    Uiso 0.0080(4)  8
O5    O2-  -0.3536(11) -0.3746(14)  0.4181(14)   1.000    Uiso 0.0080(4)  8
O6    O2-  0.4065(10)  -0.3537(11)  -0.3581(10)  1.000    Uiso 0.0080(4)  8
```

Orthorhombic @293K

```
# phase info for Mn2 O3-ortho follows
_pd_phase_name  "Mn2 O3-ortho"
_cell_length_a  9.403868
_cell_length_b  9.423538
```

```
_cell_length_c  9.414502
_cell_angle_alpha  90
_cell_angle_beta   90
_cell_angle_gamma  90
_cell_volume  834.292
_symmetry_cell_setting  orthorhombic
_symmetry_space_group_name_H-M  "P c a b"
loop_
    _space_group_symop_id
    _space_group_symop_operation_xyz
     1  x,y,z
     2  1/2-x,y,1/2+z
     3  1/2+x,1/2-y,z
     4  -x,1/2-y,1/2+z
     5  -x,-y,-z
     6  1/2+x,-y,1/2-z
     7  1/2-x,1/2+y,-z
     8  x,1/2+y,1/2-z

# ATOMIC COORDINATES AND DISPLACEMENT PARAMETERS
loop_
   _atom_site_label
   _atom_site_type_symbol
   _atom_site_fract_x
   _atom_site_fract_y
   _atom_site_fract_z
   _atom_site_occupancy
   _atom_site_adp_type
   _atom_site_U_iso_or_equiv
   _atom_site_symmetry_multiplicity
Mn1    Mn3+ 0.00000     0.00000      0.00000     1.000      Uiso 0.0031(11) 4
Mn2    Mn3+ 0.00000     0.00000      0.50000     1.000      Uiso 0.0059(12)  4
Mn3    Mn3+ 0.28412     0.25595     -0.00880     1.000      Uiso 0.0055(3)   8
Mn4    Mn3+ 0.00793     0.28540      0.24399     1.000      Uiso 0.0059(4)    8
Mn5    Mn3+ 0.25377     0.00033      0.28551     1.000      Uiso 0.0047(4)    8
O1     O2-    0.13778   -0.08639      0.16064     1.000      Uiso 0.033(3)      8
O2     O2-    0.14825    0.13013     -0.07323     1.000      Uiso 0.0105(28)  8
O3     O2-   -0.08224    0.14640      0.12151     1.000      Uiso 0.0158(31)  8
O4     O2-   -0.37786    0.41340    -0.35593     1.000      Uiso 0.0031(23)  8
O5     O2-   -0.35405   -0.37603     0.41652     1.000      Uiso -0.0023(24) 8
O6     O2-    0.40812   -0.35206    -0.35845     1.000      Uiso 0.0103(28)  8
```

Cubic @300K

```
# phase info for Mn2 O3-cubic follows
_pd_phase_name  "Mn2 O3-cubic"
_cell_length_a  9.413694
_cell_length_b  9.413694
_cell_length_c  9.413694
_cell_angle_alpha  90
_cell_angle_beta   90
_cell_angle_gamma  90
_cell_volume  834.219
_symmetry_cell_setting  cubic
_symmetry_space_group_name_H-M  "I a -3"
loop_
```

```
  _space_group_symop_id
  _space_group_symop_operation_xyz
   1  x,y,z
   2  z,x,y
   3  y,z,x
   4  1/2+x,y,1/2-z
   5  1/2-z,1/2+x,y
   6  y,1/2-z,1/2+x
   7  -z,1/2+x,1/2-y
   8  1/2-y,-z,1/2+x
   9  1/2+y,1/2-z,-x
  10  -x,1/2+y,1/2-z
  11  1/2-z,-x,1/2+y
  12  1/2+x,1/2-y,-z
  13  -x,-y,-z
  14  -z,-x,-y
  15  -y,-z,-x
  16  1/2-x,-y,1/2+z
  17  1/2+z,1/2-x,-y
  18  -y,1/2+z,1/2-x
  19  z,1/2-x,1/2+y
  20  1/2+y,z,1/2-x
  21  1/2-y,1/2+z,x
  22  x,1/2-y,1/2+z
  23  1/2+z,x,1/2-y
  24  1/2-x,1/2+y,z
  25  1/2+x,1/2+y,1/2+z
  26  1/2+z,1/2+x,1/2+y
  27  1/2+y,1/2+z,1/2+x
  28  x,1/2+y,-z
  29  -z,x,1/2+y
  30  1/2+y,-z,x
  31  1/2-z,x,-y
  32  -y,1/2-z,x
  33  y,-z,1/2-x
  34  1/2-x,y,-z
  35  -z,1/2-x,y
  36  x,-y,1/2-z
  37  1/2-x,1/2-y,1/2-z
  38  1/2-z,1/2-x,1/2-y
  39  1/2-y,1/2-z,1/2-x
  40  -x,1/2-y,z
  41  z,-x,1/2-y
  42  1/2-y,z,-x
  43  1/2+z,-x,y
  44  y,1/2+z,-x
  45  -y,z,1/2+x
  46  1/2+x,-y,z
  47  z,1/2+x,-y
  48  -x,y,1/2+z

# ATOMIC COORDINATES AND DISPLACEMENT PARAMETERS
loop_
  _atom_site_label
  _atom_site_type_symbol
  _atom_site_fract_x
```

```
   _atom_site_fract_y
   _atom_site_fract_z
   _atom_site_occupancy
   _atom_site_adp_type
   _atom_site_U_iso_or_equiv
   _atom_site_symmetry_multiplicity
Mn1    Mn3+ 0.00000         0.00000         0.00000           1.000     Uiso 0.00513(23) 8
Mn2    Mn3+ 0.28505(4)      0.00000         0.25000           1.000     Uiso 0.00536(15) 24
O1     O2-     0.12938(17)   0.14886(18)  -0.08348(15)      1.000     Uiso 0.0138(4)  48
```

Cubic @330K

```
# phase info for Mn2 O3-cubic follows
_pd_phase_name  "Mn2 O3-cubic"
_cell_length_a  9.414777
_cell_length_b  9.414777
_cell_length_c  9.414777
_cell_angle_alpha  90
_cell_angle_beta   90
_cell_angle_gamma  90
_cell_volume  834.507
_symmetry_cell_setting  cubic
_symmetry_space_group_name_H-M  "I a -3"
loop_
    _space_group_symop_id
    _space_group_symop_operation_xyz
     1  x,y,z
     2  z,x,y
     3  y,z,x
     4  1/2+x,y,1/2-z
     5  1/2-z,1/2+x,y
     6  y,1/2-z,1/2+x
     7  -z,1/2+x,1/2-y
     8  1/2-y,-z,1/2+x
     9  1/2+y,1/2-z,-x
    10  -x,1/2+y,1/2-z
    11  1/2-z,-x,1/2+y
    12  1/2+x,1/2-y,-z
    13  -x,-y,-z
    14  -z,-x,-y
    15  -y,-z,-x
    16  1/2-x,-y,1/2+z
    17  1/2+z,1/2-x,-y
    18  -y,1/2+z,1/2-x
    19  z,1/2-x,1/2+y
    20  1/2+y,z,1/2-x
    21  1/2-y,1/2+z,x
    22  x,1/2-y,1/2+z
    23  1/2+z,x,1/2-y
    24  1/2-x,1/2+y,z
    25  1/2+x,1/2+y,1/2+z
    26  1/2+z,1/2+x,1/2+y
    27  1/2+y,1/2+z,1/2+x
    28  x,1/2+y,-z
```

```
 29  -z,x,1/2+y
 30  1/2+y,-z,x
 31  1/2-z,x,-y
 32  -y,1/2-z,x
 33  y,-z,1/2-x
 34  1/2-x,y,-z
 35  -z,1/2-x,y
 36  x,-y,1/2-z
 37  1/2-x,1/2-y,1/2-z
 38  1/2-z,1/2-x,1/2-y
 39  1/2-y,1/2-z,1/2-x
 40  -x,1/2-y,z
 41  z,-x,1/2-y
 42  1/2-y,z,-x
 43  1/2+z,-x,y
 44  y,1/2+z,-x
 45  -y,z,1/2+x
 46  1/2+x,-y,z
 47  z,1/2+x,-y
 48  -x,y,1/2+z

# ATOMIC COORDINATES AND DISPLACEMENT PARAMETERS
loop_
  _atom_site_label
  _atom_site_type_symbol
  _atom_site_fract_x
  _atom_site_fract_y
  _atom_site_fract_z
  _atom_site_occupancy
  _atom_site_adp_type
  _atom_site_U_iso_or_equiv
  _atom_site_symmetry_multiplicity
Mn1   Mn3+  0.00000      0.00000      0.00000       1.000   Uiso 0.00759(22) 8
Mn2   Mn3+  0.28502(4)   0.00000      0.25000       1.000   Uiso 0.00814(14) 24
O1    O2-   0.13019(16)  0.14859(17)  -0.08346(14)  1.000   Uiso 0.0121(4)  48
```

III. Description of the electrode preparation, conductivity measurements and electrochemical measurements.

For electrochemical measurements 5 milligrams of the α-Mn_2O_3 prism sample was mixed with 2 mL ethanol, 3 mL ultrapure water, and 50 µL Nafion (5 wt%). This solution was sonicated for 30 minutes to generate a homogeneous ink. Then 5 µL of the catalyst ink was loaded onto the polished surface of a 4-mm diameter interchangeable glassy carbon electrode disk (GCE; ALS Co. Part #: 013362) and dried slowly at room temperature to achieve a uniform surface. Once the sample had completely dried, it was weighed on an analytical balance to estimate the mass of α-Mn_2O_3 prisms delivered to the surface. The typical weight was 5 ± 0.21 µg (i.e. 40 ± 0.21 µg cm^{-2}) and had uniform coverage according to previous accounts.[1] To prepare the α-Mn_2O_3 prisms for electrochemical measurements, the GC disk was heated to 480 °C in air for 10 hours.

Then the electrode assembly was reassembled by replacing the GC with the α-Mn_2O_3 prism sample pointing outward. Since the 480 °C heating step might affect the GC electrode, we examined the LSV curves of the bare GC before and after heat treatment at 480 °C and there was very little change, indicating that oxidative damage was negligible.[1] 20 wt% Pt/C catalyst was used as comparison and prepared exactly as the synthesized prisms to achieve a similar loading of 5 ± 0.25 μg of 20 wt% Pt/C (i.e. 40 ± 0.25 μg cm^{-2}). The Pt/C sample was measured immediately after drying at room temperature. A similar procedure was used to make the RuO_2 electrode.

The conductivities were measured by the AC impedance method using a Hokuto Denko HZ7000 electrochemical instrument in the frequency range of 100 Hz to 1 Hz at an AC amplitude of 100 mV, and then the conductivity values at 1 Hz were plotted. The powder sample was loaded between two titanium electrodes (5 mmϕ) of a metallic cell. The powder was compressed with springs (thickness of the sample: 0.309 mm). After running AC impedance measurements in the temperature range from 20°C to 50°C for two days (conditioning), the sample loaded on the cell was dried at 80°C under vacuum for a few days. Then, the conductivity was evaluated in the temperature range from 0°C to 50°C in a noise-free Peltier module chamber with a temperature step of 2.5 °C.

All measurements were carried out at room temperature using a potentiostat (Biologic VMP3) with a 3-electrode electrochemical cell. The interchangeable GCE with a 4 mm diameter (area ~0.125 cm^2) functioned as the working electrode, a platinum (Pt) wire as the counter electrode, and Hg/HgO (1 M NaOH) as the reference electrode. The potential of the reference electrode (Hg/HgO, 1 M NaOH) in 0.1 M KOH shifts to 0.870 V vs. RHE based on our calculations, which agrees with the value reported in the literature. Linear sweep voltammetry (LSV) measurements were performed in O_2-saturated 0.1 M KOH solutions at a scan rate of 10 mV s^{-1}. LSVs were measured using forward and backward currents to confirm the reversibility of the catalysts.[1] Exchange current density (i_0) was extracted from the Tafel diagram (overpotential vs log(i)) by extrapolating the linear region to 1.23V. The LSVs for each reaction were run 10-times at each temperature to generate the error bars in Figure 4. Changing temperature will generate negligible change in the pH of the KOH solution because it is a strong base. By our estimation it will change pH by ~0.07 which will generate a maximum downward shift in the Nernst potential

(in ORR) of only 4 mV. In addition, the temperature-dependent change in the specific conductivity of an aqueous 0.1 M KOH solution has been experimentally shown to be linear.[2] This indicates that any superlinear increase in exchange current density would be due to the catalyst/electrode system. LSVs were performed 10-times at each temperature.

IV. Reference

(1) Jahan, M.; Tominaka, S.; Henzie, J. Phase Pure α-Mn2O3 Prisms and Their Bifunctional Electrocatalytic Activity in Oxygen Evolution and Reduction Reactions. *Dalt. Trans.* **2016**, *45* (46), 18494–18501.

(2) Gilliam, R. J.; Graydon, J. W.; Kirk, D. W.; Thorpe, S. J. A Review of Specific Conductivities of Potassium Hydroxide Solutions for Various Concentrations and Temperatures. *Int. J. Hydrogen Energy* **2007**.
